# *Drosophila melanogaster* Models of Friedreich's Ataxia

**DOI:** 10.1155/2018/5065190

**Published:** 2018-04-05

**Authors:** P. Calap-Quintana, J. A. Navarro, J. González-Fernández, M. J. Martínez-Sebastián, M. D. Moltó, J. V. Llorens

**Affiliations:** ^1^Department of Genetics, University of Valencia, Campus of Burjassot, Valencia, Spain; ^2^Institute of Zoology, University of Regensburg, Regensburg, Germany; ^3^Biomedical Research Institute INCLIVA, Valencia, Spain; ^4^Centro de Investigación Biomédica en Red de Salud Mental (CIBERSAM), Madrid, Spain

## Abstract

Friedreich's ataxia (FRDA) is a rare inherited recessive disorder affecting the central and peripheral nervous systems and other extraneural organs such as the heart and pancreas. This incapacitating condition usually manifests in childhood or adolescence, exhibits an irreversible progression that confines the patient to a wheelchair, and leads to early death. FRDA is caused by a reduced level of the nuclear-encoded mitochondrial protein frataxin due to an abnormal GAA triplet repeat expansion in the first intron of the human* FXN* gene.* FXN* is evolutionarily conserved, with orthologs in essentially all eukaryotes and some prokaryotes, leading to the development of experimental models of this disease in different organisms. These FRDA models have contributed substantially to our current knowledge of frataxin function and the pathogenesis of the disease, as well as to explorations of suitable treatments*. Drosophila melanogaster*, an organism that is easy to manipulate genetically, has also become important in FRDA research. This review describes the substantial contribution of* Drosophila* to FRDA research since the characterization of the fly frataxin ortholog more than 15 years ago. Fly models have provided a comprehensive characterization of the defects associated with frataxin deficiency and have revealed genetic modifiers of disease phenotypes. In addition, these models are now being used in the search for potential therapeutic compounds for the treatment of this severe and still incurable disease.

## 1. Introduction

Friedreich's ataxia (FRDA) is an autosomal recessive neurodegenerative disorder and the most common form of hereditary ataxia among populations of European origin (2–4/100,000) [1]. This disabling condition typically manifests before age 25, with progressive neurodegeneration of the dorsal root ganglia, sensory peripheral nerves, corticospinal tracts, and dentate nuclei of the cerebellum. A large proportion of patients develop hypertrophic cardiomyopathy, which is the major cause of reduced life expectancy in this disease. Diabetes mellitus and impaired glucose tolerance are also seen in a significant number of FRDA patients (reviewed in [2]).

FRDA is caused by loss-of-function mutations in the* FXN* gene, which encodes the frataxin protein [3]. Frataxin is a small protein encoded in the nucleus, expressed as a precursor polypeptide in the cytoplasm and imported into mitochondria [4–6]. The majority of FRDA patients are homozygous for an abnormally expanded GAA repeat in intron 1 of* FXN*, resulting in strongly reduced frataxin protein expression (from 5% to 30% of the normal level) [7]. The remaining FRDA patients are compound heterozygotes, carrying the GAA repeat expansion on one* FXN* allele and another pathogenic mutation on the other allele, including point mutations and insertion and/or deletion mutations [8].

**Figure 1:**
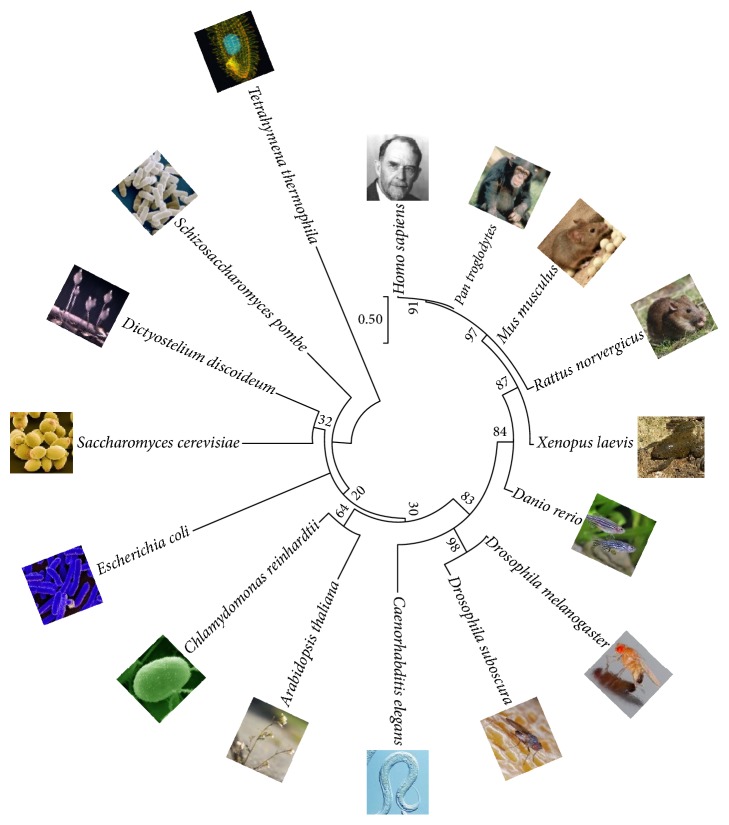
Molecular phylogenetic analysis of frataxin sequences from different species. The picture of Thomas Hunt Morgan was chosen to represent* Homo sapiens* because, as a result of his work,* D. melanogaster* became a major model organism in genetics. Methods: evolutionary history was inferred with the maximum likelihood method based on Le and Gascuel model [9]. The tree with the highest log likelihood (−2026.7976) is shown. Initial trees for the heuristic search were obtained automatically by applying the Neighbor-Joining and BioNJ algorithms to a matrix of pairwise distances estimated using a JTT model and then selecting the topology with the superior log likelihood value. A discrete gamma distribution was used to model evolutionary rate differences among sites (5 categories (+G, parameter = 2.4842)). The tree is drawn to scale, with branch lengths representing the number of substitutions per site. The analysis involved 16 amino acid sequences. All positions containing gaps and missing data were eliminated. A total of 90 positions were present in the final dataset. Evolutionary analyses were conducted in MEGA7 [10].

A lack of available patients and the inherent limitations of cellular models often hinder the discovery and detailed analyses of genes and pathways relevant to the pathology of rare human disorders such as FRDA. Fortunately, the high evolutionary conservation of frataxin (Figure 1) has enabled the development of disease models in several organisms, from bacteria to mice, that have significantly contributed to the understanding of frataxin function. The development of these disease models is an essential step in elucidating underlying pathological mechanisms and identifying efficient treatments in FRDA.

Seminal findings reported by key studies in model organisms (reviewed in [14–23]) have suggested potential roles for frataxin in iron homeostasis and cellular defense against reactive oxygen species (ROS), as an activator of the mitochondrial respiratory chain, as a mitochondrial chaperone, and as a regulator of Fe-S cluster (ISC) assembly. Although frataxin function is not yet fully characterized, its role in ISC biogenesis is generally accepted [24–26]. Major alterations associated with frataxin deficiency include mitochondrial iron accumulation, oxidative stress hypersensitivity, impaired ISC biogenesis, and aconitase and respiratory chain dysfunction (reviewed in [27–29]).

Although the arthropod lineage diverged from the vertebrate lineage more than 600 MYA, genome sequencing projects have revealed a large number of biological processes that are conserved between flies and vertebrates. Most of the genes implicated in familial forms of disease have at least one* Drosophila* ortholog [30, 31]. This species offers many different genetic tools that can be applied to investigate basic biological questions in a multicellular organism, with the advantages of easy manipulation and culture.

## 2. The* Drosophila* Ortholog of the* FXN* Gene

The* D. melanogaster *frataxin ortholog was cloned and characterized in our laboratory in the early 2000s. It was named* dfh (Drosophila frataxin homolog)* [32]. This gene is referred to as* fh (frataxin homolog)* in FlyBase (CG8971, FBgn0030092), and this name will be used throughout this review. We isolated* fh* by screening a genomic library from* D. subobscura* using human* FXN* probes. Database searches employing the sequence of* D. subobscura* positive clones led to the identification of the* D. melanogaster* STS 125a12, mapped to the 8CD region on the X chromosome and cloned in cosmid 125a12. Further characterization of this cosmid showed an open reading frame (ORF) encoding a frataxin-like protein. Screening of an adult cDNA library from* D. melanogaster, *using the genomic frataxin ORF, revealed two transcripts with two different polyadenylation signals. We confirmed that this gene is located in the 8CD region by in situ hybridization analysis of polytene chromosomes of* D. melanogaster* using* fh* cDNA as a probe.

**Figure 2:**
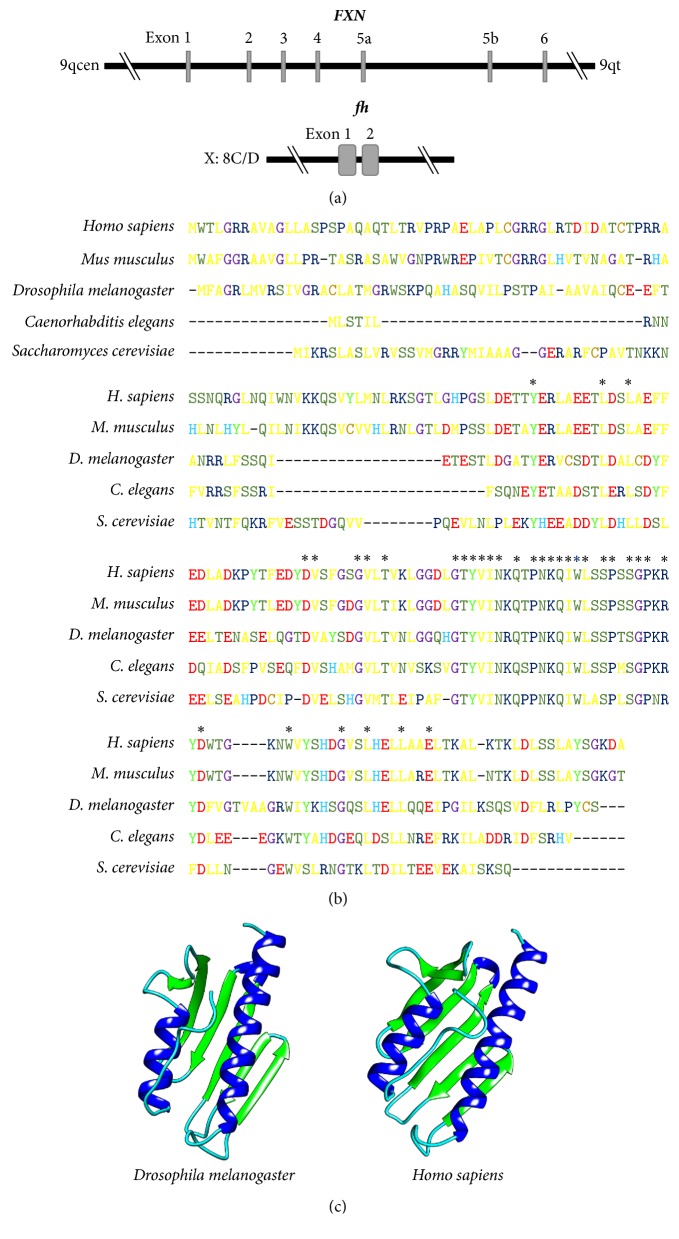
The* Drosophila* frataxin ortholog. (a) Genomic organization of the human* (FXN)* and the fly* (fh)* genes encoding frataxin.* FXN* is located in 9q21.11 and contains seven exons.* fh* is located in chromosome X: 8C14 and has two exons. (b) Multiple alignment of the frataxin protein sequences of* Homo sapiens*,* Mus musculus*,* D. melanogaster*,* Caenorhabditis elegans*, and* Saccharomyces cerevisiae*. The letters indicate the amino acid in each position, and the colors classify the amino acids according to their biochemical properties, as described in the MEGA7 program [10]. Invariant amino acids are marked with an asterisk. (c) The 3D structure prediction of the frataxin protein using the Phyre 2 [11] and Chimera 1.12 software [12]; *α*-helixes appear in blue and *β*-sheets in green.

The genomic organization of* fh *is much simpler than that of the human gene (Figure 2(a)) [32].* fh* is approximately 1 kb and is composed of two exons of 340 bp and 282 bp, separated by an intron of 69 bp. RNA in situ hybridization in whole embryos showed ubiquitous expression of* fh* in all developmental stages examined (from 2 to 16 h). ~1 kb major transcript was identified by Northern blot analysis, in agreement with the predicted size of one of the two mRNA sequences detected by cDNA library screening. This transcript was found in embryonic, larval, pupal, and adult stages [32]. Accordingly, the protein was present in all developmental stages at varying levels, reaching its highest level in late embryos [33].

The encoded fly protein was predicted to have 190 amino acids, with a molecular weight of ~21 kDa. A sequence comparison of frataxin proteins from different species showed better alignment in the central and the C-terminal regions (Figure 2(b)), whereas no alignment was found in the N-terminal region of the protein. Importantly, this region of fly frataxin (FH) also showed typical frataxin features, such as a mitochondrial signal peptide and a putative *α*-helix with abundant positively charged amino acids and few negatively charged residues [32]. Colocalization experiments using an FH-enhanced green fluorescent fusion protein (EGFP) and a mitochondrial marker confirmed the localization of FH in mitochondria [34]. The mature form of FH has a molecular weight of ~15 kDa [33]. The secondary structure of FH matches the *α*-*β* sandwich motif characteristic of other frataxin proteins encoded by orthologous genes [32]. Predictions of the 3D structure generated using the Phyre 2 [11] and Chimera 1.12 [12] software show that FH has an organization similar to that of the human protein (Figure 2(c)). The biophysical properties of FH indicate that its thermal and chemical stabilities closely resemble those of human frataxin [35]. Unlike other eukaryotic frataxin proteins, FH shows enhanced stability* in vitro*, making it a more attractive candidate for evaluation of metal binding and delivery properties. In these experimental conditions, FH can bind and deliver Fe(II), which is required for ISC biosynthesis [35], and, as previously described for human frataxin [36], it interacts with Isu (the Fe cofactor assembly platform for ISC cellular production) in an iron-dependent manner [35]. Recently, some authors have provided experimental evidence that the initial complex of the mitochondrial ISC biosynthetic machinery is conserved in* Drosophila* [37, 38]. These results, along with those reported in mouse (reviewed in [39]), suggest an evolutionarily conserved role for frataxin in ISC biosynthesis.

## 3. Modeling FRDA in Flies

**Figure 3:**
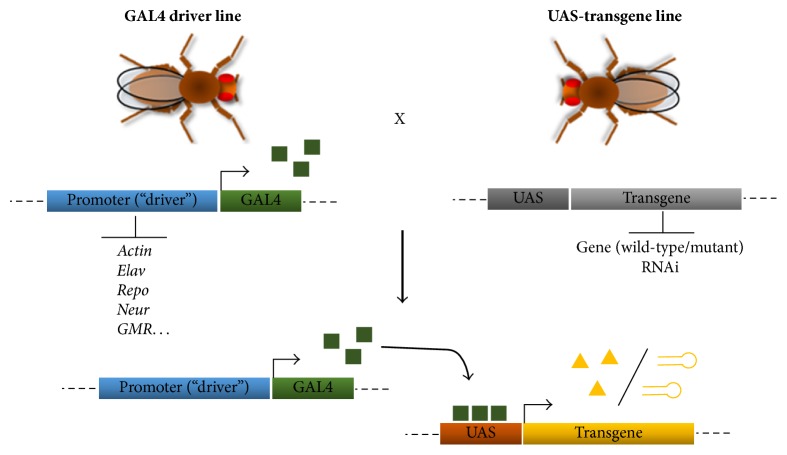
The GAL4/UAS system, adapted from yeast, involves the use of two transgenic lines in* Drosophila *[13]. One line carries the GAL4 transcription factor under the control of a promoter of known expression pattern (the driver line), and the other line contains the transgene of interest downstream of UAS (the responder line). Many GAL4 driver lines are available, carrying the promoters of genes such as* actin* (ubiquitous),* elav* (pan-neuronal),* repo* (glial cells),* neur* (sensory organs), and* GMR* (eye). This system is very versatile and allows the expression of specific genes or gene constructs to be induced or suppressed. Triangles indicate a wild-type or mutant protein; the hairpins represent double-stranded RNA molecules that mediate RNAi.

Several models of FRDA have been developed in* D. melanogaster*, mainly taking advantage of GAL4/UAS transgene-based RNA interference (RNAi) methodology. RNAi allows the posttranscriptional silencing of a gene via the expression of transgenic double-stranded RNAs [40]. The GAL4/UAS system [13] has been incredibly successful in* D. melanogaster* and can induce the expression of a transgene under the control of UAS (Upstream Activating Sequences) and the transcriptional activator protein GAL4 (Figure 3). This experimental strategy has been used to induce tissue-specific and ubiquitous knockdown of* fh* (Table 1). Therefore, this strategy allows the phenotypes of FRDA patients to be mimicked by reducing rather than completely eliminating FH.

The first UAS-transgene construct for RNAi-mediated silencing of* fh* expression was reported by Anderson et al. [33]. This construct consisted of inverted repeats containing the first 391 nucleotides of the* fh* coding region, which were subcloned into the pUAST vector. Fly transformants were crossed to the *da*^G32^ GAL4-driver line (which exhibits widespread GAL4 protein expression throughout development and in most tissues under the control of regulatory sequences of* daughterless*) to examine* fh* silencing. Three transgenic lines (UDIR1, UDIR2, and UDIR3) were selected in which the GAL4-regulated transgene substantially reduced the FH protein level [33, 41]. Similarly, Llorens et al. [34] generated another UAS-transgene construct (named UAS-*fh*IR) containing two copies of the* fh* coding region in opposite orientations, separated by a GFP fragment as a spacer. A transgenic line (*fh*RNAi line) was selected showing milder effect than the GAL4-regulated transgene in UDIR1/2/3 when crossed with the *da*^G32^ GAL4 line (Table 1).

The RNAi lines from John Phillips's laboratory [33] have also been combined with a ligand-inducible GAL4/UAS system to deplete frataxin in the* Drosophila* heart [42]. This system is based on a steroid-activated chimeric GAL4 protein, specifically the GAL4-progesterone-receptor fusion protein that is activated by RU486 (mifepristone) [43, 44]. Transgene expression is induced by supplementing the fly food with RU486, and the level of expression is controlled by changing the dosage of the steroid ligand [43].

More recently, Chen et al. [45] identified the first mutant allele of* fh (fh*^1^) in an unbiased genetic screen of the X chromosome designed to isolate mutations that cause neurodegenerative phenotypes. The mutant allele consisted of an ethyl-methanesulfonate-induced missense mutation (S136R) located in a highly conserved region (S157 in the human protein) required for the binding of human frataxin to the ISC assembly complex [45, 46]. The authors also generated mosaic* fh* mutant mitotic clones of adult photoreceptor neurons using the eyeless-FLP/FRT system to bypass the lethality associated with the* fh*^1^ mutation [45].

These* Drosophila* models of FRDA have been employed to study frataxin function, analyze conserved pathological mechanisms, and search for genetic modifiers and potential therapies. The main results of such studies are described in the following sections.

## 4. Phenotypes of Frataxin Deficiency in* Drosophila*

The loss of* fh* function in* Drosophila* recapitulates important biochemical, cellular, and physiological phenotypes of FRDA. In addition, some phenotypes have been described for the first time in this organism, revealing new key players in FRDA pathogenesis. All these phenotypes have been obtained using the* fh* constructs and alleles that were described above. Table 1 details these features as well as the temperature of the crosses when available, because the GAL4/UAS system is sensitive to this parameter.

Near-complete frataxin depletion in* Drosophila* seriously affects viability, similar to observations in the FRDA mouse model [47] and most likely in humans, since no patients carrying a pathogenic point mutation or deletion or insertion mutations in both* FXN* alleles have been reported. Ubiquitous* fh* suppression affects larva and pupa development, and individuals do not reach the adult phase [33, 34]. In agreement with these results, individuals that are hemizygous for the* fh*^1^ mutant, carrying the missense S136R mutation, show lethality from the instar 3 larva to pupa stages [45]. Silencing of* fh* in developing muscle and heart tissue (using the* 24B* and* Dot* driver lines) is also lethal in pupal stages, while reduction of* fh* expression in subsets of neurons (C96,* Ddc*,* D42*,* c698a*, and* neur*) allows the development of viable adults. Importantly, when* fh* expression is specifically reduced in the peripheral nervous system (PNS), using the* C96* and* neur* GAL4 lines, the adult flies show a shortened lifespan and reduced climbing ability [33, 34]. These results indicated that, in* Drosophila*, as in humans, frataxin is an essential protein and that different tissues have distinct sensitivity to frataxin deficiency.

Tricoire et al. [42] obtained the first fly* in vivo* heart images after heart-specific depletion of frataxin using the UDIR2 line and the RU486-inducible Geneswitch driver HandGS. They observed major cardiac dysfunction including impaired systolic function and substantial heart dilatation, resembling the phenotypes observed in FRDA patients. The cellular neuropathology of frataxin deficiency was examined in larval motor neurons using the UDIR1 line [48]. Loss of mitochondrial membrane potential was detected in the cell bodies, axons, and neuromuscular junction of segmental nerves from second to late third instar larvae. These effects were followed by defects in mitochondrial retrograde transport in the distal axons, leading to a concomitant dying-back neuropathy. A dying-back mechanism has also been described in sensory neurons and the spinocerebellar and corticospinal motor tract in patients (reviewed in [29]).

To more closely mimic the patient situation, viable adults with ubiquitous reduction of FH were obtained by Llorens et al. [34] by crossing the* fh*RNAi line with the actin-GAL4 driver at 25°C. Under these experimental conditions, the* fh* mRNA level was reduced to one-third compared with the normal level. As in humans [7], the remaining frataxin (approximately 30% of the normal level) allowed normal embryonic development but resulted in decreased lifespan and impaired motor performance in adulthood. Specifically, survival analysis showed a decrease of 60% and 32% in the mean and maximum lifespan, respectively, compared with controls. The FRDA flies showed limited climbing ability in negative geotaxis assays, with 5-day-old adults exhibiting a 45% decline compared with control flies.

Frataxin deficiency in flies also triggers iron accumulation [45, 49] restricted to mitochondria [49], consistent with findings in other model organisms and FRDA patients. Importantly, the role of iron in the pathophysiology of FRDA has not yet been completely established and is still a matter of debate. The discovery of iron deposits in the hearts of FRDA patients in the late seventies [50, 51] was the first indication of an association between frataxin and this transition metal. This relationship became more important after the discovery that the loss-of-function of the yeast frataxin ortholog results in mitochondrial iron accumulation [52]. Since then, iron-enriched granules have been further confirmed in patient hearts [53–55] and in several other patient tissues [56, 57]. Surprisingly, analyses of iron levels in neuronal tissues have shown inconsistent results, even in tissues with high frataxin expression. On the one hand, histological and imaging approaches have detected alterations in the expression of iron-related proteins that support the hypothesis that iron redistribution rather than iron accumulation is the key defect underlying frataxin deficiency in the nervous system [58, 59]. On the other hand, increased iron content has been reported in critical brain areas of FRDA patients [60, 61]. In* Drosophila,* Chen et al. showed that iron accumulates in the nervous system in* fh*^1^ mutants [45]. These authors also found increased levels of iron in the nervous system in an FRDA mouse model that exhibits less than 40% of the normal level of frataxin mRNA in this tissue [62]. By contrast, no iron deposits have been reported in the nervous system in other mouse models of FRDA [47, 63–65]. In line with the proposed iron toxicity in FRDA, all* Drosophila* models share an enhanced sensitivity to increased iron content in food [33, 45, 66].

The analysis of the iron-frataxin relationship in several FRDA models has provided experimental evidence supporting a role for frataxin in iron homeostasis (storage, redistribution, chaperone, and ISC biosynthesis, reviewed in [23, 24]). Supporting a role for frataxin in ISC assembly, loss of FH expression is associated with impaired activity of Fe-S containing enzymes, including proteins involved in the mitochondrial electron transport chain (ETC) and aconitase [33, 34]. This effect causes problems in ATP production, which is reduced in* Drosophila* models independently of the levels of functional frataxin [33, 34, 45], as well as in FRDA patients [67, 68]. In addition, the biochemical and biophysical characterization of FH is consistent with its expected role as an iron chaperone acting as a regulator during ISC biosynthesis [35]. In line with this role for frataxin, its suppression in the prothoracic gland impairs the ability of larvae to initiate pupariation [69]. This organ produces ecdysteroid hormones, such as 20-hydroxyecdysone, that mediate developmental transitions. Interestingly, some Fe-S-containing enzymes such as Neverland (converts cholesterol into 7-dehydrocholesterol) and the fly ferredoxins Fdxh and Fdxh2 participate in the metabolism of ecdysone, and their activities are likely impaired in frataxin-deficient larvae. In agreement with this hypothesis, 20-hydroxyecdysone supplementation improves the defective transitions associated with frataxin deficiency in the prothoracic gland [69]. An ecdysone deficiency would explain the giant, long-lived larvae phenotype reported by Anderson et al. in their fly model using the UDIR2 line and *da*^G32^ GAL4 driver [33]. Interestingly,* Drosophila* models have also revealed that iron deregulation occurs before the decrease in the activity of mitochondrial enzymes [49, 66]. This is in agreement with results from an inducible yeast model in which the iron regulon was activated long before decreased aconitase activity was observed [70].

It has been suggested that ROS are generated by iron accumulation through Fenton's reaction, damaging the mitochondrial ETC and mediating the pathophysiology of FRDA (reviewed in [20, 71]). However, the role of oxidative stress in the disease is still questioned, and controversial results have also been reported in* Drosophila*. Overexpression of ROS-scavenging enzymes such as catalase (CAT), superoxide dismutase 1 (SOD1), or SOD2 could not rescue the pupae lethality caused by ubiquitous UDIR1 and UDIR2 expression [33] or the photoreceptor neurodegeneration in* fh*^1^ mutant clones [45]. CAT overexpression and treatment with EUK8 (a synthetic superoxide dismutase and catalase mimetic) also failed to improve cardiac function in frataxin-depleted hearts [42]. Shidara and Hollenbeck [48] did not detect increased ROS levels in frataxin-deficient motor neurons, but these neurons responded to the complex III inhibitor antimycin A with a larger increase in ROS than control neurons.

However, increasing evidence from different FRDA models and patient samples suggests that oxidative stress is a major player in FRDA [34, 41, 65, 72–80]. In* Drosophila*, increased levels of malondialdehyde (MDA, a lipoperoxidation product) have been reported in flies with ubiquitous FH suppression using the* fh*RNAi line and the* actin* GAL4-driver line [81, 82]. These flies and flies with tissue-specific frataxin deficiency in the PNS* (C96)* or glial cells* (repo)* showed increased sensitivity to external oxidative insults (see Table 1) such as hyperoxia or H_2_O_2_ treatment [41, 81, 83]. Hyperoxia induces enhanced aconitase inactivation in the frataxin knockdown flies [34, 83], which compromises the entire respiratory process. In fact, hyperoxia leads to reduced oxygen consumption rates in mitochondrial extracts of the frataxin-depleted flies [34]. Overexpression of the H_2_O_2_-scavenging enzymes CAT, mitoCAT (using a synthetic transgene that targets CAT to the mitochondria), or mitochondrial peroxiredoxin (mTPx) rescues the shortened lifespan and increased sensitivity to H_2_O_2_ in flies with reduced frataxin expression in the PNS* (C96)* [41]. These scavengers also restore aconitase activity in flies with systemic reduction of FH using the UDIR1 line and the *da*^G32^ GAL4 driver [41], supporting the role of oxidative stress in aconitase inactivation. In addition, scavengers of lipid peroxides have been shown to improve frataxin-deficient phenotypes [83, 84].

Recently, Hugo Bellen's laboratory identified a new mechanism for neuronal degeneration in FRDA, in which iron toxicity is not associated with ROS damage [45]. These authors showed in their* fh* mutant that iron accumulation induces sphingolipid synthesis and activates the expression of the genes* 3-phosphoinositide dependent protein kinase-1 (Pdk1)* and* myocyte enhancer factor-2 (Mef2)* and their downstream targets, causing loss of photoreceptors in fly ommatidia. In agreement with these results, inhibition of sphingolipid synthesis by downregulating the expression of the rate-limiting enzyme lace (the fly ortholog of serine palmitoyltransferase) or feeding the mutant flies Myriocin (a compound that inhibits serine palmitoyltransferase) was sufficient to partially revert the cellular degeneration [45]. Similarly, silencing* Pdk1* or* Mef2* expression also suppressed the neurodegenerative phenotype. Remarkably, the authors found that loss of frataxin in the nervous system in mice and in heart tissue from patients also activates the same pathway, suggesting a conserved mechanism [62]. These results highlight, once more, the relevance of* Drosophila* in the study of human disorders such as FRDA. In addition, they strongly suggest that iron plays an instrumental role in* Drosophila* frataxin biology.

Similarly,* Drosophila* has also been a pioneer model organism in highlighting the role of frataxin in lipid homeostasis [83]. Ubiquitous frataxin knockdown or targeted frataxin downregulation in glia cells triggered lipid accumulation. Increased amounts of myristic acid (C14:0), palmitic acid (C16:0), palmitoleic acid (C16:1), oleic acid (C18:1), and linoleic acid (C18:2) were found. These results suggested that loss of mitochondrial function also affects fatty acid beta-oxidation, leading to the accumulation of the most abundant lipid species [83]. The presence of lipid droplets had already been characterized in mouse models [63], and the fly findings indicated the content of these droplets and their likely association with the disease pathophysiology. These findings were followed by assessments of lipid deregulation in other models [85] and in patient samples [86]. The association between frataxin and lipid metabolism has been extensively reviewed elsewhere [87].

## 5. Frataxin Overexpression Phenotypes

Although frataxin overexpression does not model the disease, it is an excellent complementary tool to further describe the cellular roles of frataxin. In this regard,* Drosophila* models have shown that some increase in frataxin expression is beneficial, whereas its excess beyond certain thresholds is clearly detrimental.  Table 2 summarizes the phenotypes reported for frataxin overexpression in flies using several GAL4 lines that drive ubiquitous or tissue-specific* fh* expression.

Flies with ubiquitous* fh* expression at a level approximately fourfold higher than the physiological level show increased longevity, antioxidant defense responses, and resistance to treatment with paraquat (a chemical known to specifically affect mitochondrial complex I and to generate free radicals), H_2_O_2_, and dietary iron [89]. Similarly, it has been reported that frataxin overexpression in mice [90, 91] or in cultured cells [92–94] is innocuous or has a positive effect, stimulating ATP production or inducing antioxidant defense responses.

A systemic 9-fold increase in* fh* mRNA expression impairs muscle, heart, and PNS development in fly embryos, leading to lethality from larva to pupa stages [34]. Frataxin overexpression restricted to developing heart and muscle tissue (*Dot*,* 24B*; Table 2) also has deleterious effects [34]. In contrast, overexpressing FH pan-neuronally* (Appl, elav)*, in sensory organs* (neur)*, motor neurons* (D42)*, and glial cells* (repo)* produces viable adults, but they show a reduced lifespan and decreased locomotor performance [34, 95]. The effect of human frataxin expression has also been tested in* Drosophila*. FXN is correctly expressed and targeted to mitochondria in flies and can rescue the aconitase activity of UDIR2-knockdown flies [95]. These results provide* in vivo* evidence that human and fly frataxins have conserved functions, which was further confirmed by Tricoire et al. [42] and Chen et al. [45]. As expected, FXN overexpression in flies produces similar but slightly stronger phenotypes at biochemical, physiological, and developmental levels than those observed in flies overexpressing FH [95]. Initially, it was proposed that frataxin overexpression might act as a dominant negative mutation and that its toxic effect might be mediated by oxidative stress [95]. The mechanism underlying frataxin overexpression has recently been further investigated [96]. In this study, the authors reported that frataxin overexpression increases oxidative phosphorylation and modifies iron homeostasis. Such an increase of mitochondrial activity alters mitochondrial morphology and sensitizes cells to oxidative damage leading to neurodegeneration and cell death. Importantly, authors found that iron was a pivotal factor in the neurodegeneration [96].

These results in* Drosophila* show that frataxin requires an optimal balance in expression to function properly and that control of its expression is important in treatments that aim to increase its protein level.

## 6. Genetic Modifiers of FRDA

**Figure 4:**
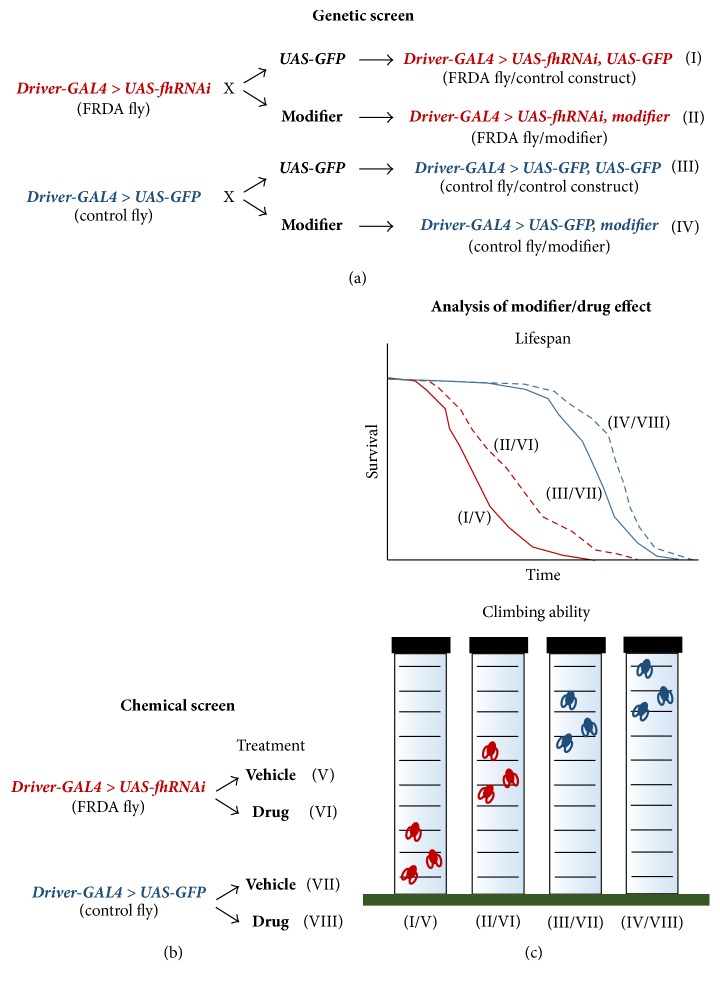
Schematic design of a genetic (a) or chemical (b) screen to identify genetic modifiers or potential therapeutic compounds in FRDA using* Drosophila* as a model organism. The effect of a genetic modifier or drug is evaluated by monitoring the lifespan and climbing ability of FRDA flies. (c) A UAS-GFP construct is included in this strategy as an internal control to determine whether the drug can interfere with the GAL4/UAS system and the potential dilution of the GAL4 protein due to the presence of two UAS construct. In parallel, the effect of the modifier or drug treatment is analyzed in control flies to identify frataxin interactors. GFP: green fluorescent protein. Vehicle: DMSO/H_2_O depending on the drug solubility.

*Drosophila* models are important because they offer the ability to carry out genetic screens for mutations that affect a particular biological process. This powerful tool provides a way to identify genetic modifiers of human diseases (Figures 4(a) and 4(c)). Our group has collaborated with Juan Botas's laboratory in two studies using this methodology in* Drosophila* models of FRDA. These studies followed a biased candidate approach, selecting genes related to disease pathophysiology [81, 82]. We set out to test whether genetic modification of key pathways would improve FRDA phenotypes in flies. Candidate genes were selected from pathways involved in metal homeostasis, the response to oxidative stress, apoptosis, and autophagy. Approximately 300 lines were analyzed, including RNAi lines from the Vienna* Drosophila* Resource Center and loss-of-function and overexpression lines from the Bloomington Stock Center (Indiana University). The external eye morphology and motor performance of adult flies were used as screening phenotypes. The UDIR2 line [33] (with a 90% reduction in FH expression when expressed ubiquitously) produces a mild rough eye phenotype when expressed in the developing eye [82]. The* fh*RNAi line [34] (with a 70% reduction in FH expression that is compatible with normal development) impairs motor performance when expressed ubiquitously. We applied a tiered strategy to examine the effect of metal-related genes on eye morphology, followed by the effect of eye modifiers on motor performance [82]. In Calap-Quintana et al. [81], we reported the effect of the remaining candidate genes on the motor performance of the* fh*RNAi line.

Five suppressors of both the eye and motor performance phenotypes were identified: the iron regulatory proteins encoded by the genes* Irp-1A* and* Irp-1B*, their target Transferrin (*Tsf1* and* Tsf3*), and* Malvolio (Mvl)*, the* Drosophila* ortholog of the mammalian gene* Divalent metal transporter-1 (DMT1)*. The suppression of these FRDA phenotypes was mediated by reducing the iron abundance associated with frataxin deficiency [82]. On the one hand, reduced expression of* Mvl*,* Tsf1*, and* Tsf3* decreases cellular iron uptake, which in turn reduces mitochondrial iron accumulation. On the other hand, downregulation of* Irp-1A* and* Irp-1B* reduces IRP activity, as suggested in [33, 66], and thus recovers ferritin expression and normal cellular iron distribution. In agreement with these findings,* Irp1* knockout reduces mitochondrial iron accumulation in frataxin-depleted mouse livers [97].

Another iron player that can suppress FRDA phenotypes in flies was identified by Navarro et al. [66]. It is a member of the mitochondrial solute carrier family named mitoferrin (Mfrn), which is located in the inner mitochondrial membrane, and its function is to translocate iron into mitochondria [98–100]. Downregulation of* mfrn* was sufficient to improve iron metabolism in frataxin-deficient flies and to ameliorate neurodegeneration triggered by targeted frataxin silencing in glia cells [66]. In this study, overexpression of ferritin subunits was unable to counteract neurodegeneration, whereas another study reported that ferritin overexpression had a positive effect in* fh* mutant clones of fly photoreceptors [45]. It is likely that the different metabolic requirements of each cell type might be reflected in the factors that can exert protective roles.

Knockdown of zinc transporters and copper chaperones also ameliorates FRDA phenotypes in flies [82]. Members of the two conserved gene families of zinc transporters (the ZnT and Zip families) improve the eye and motor performance phenotypes by normalizing iron levels in some cases. It has been previously reported that several members of the Zip family can also transport iron in addition to zinc [101–103]. Genetic reduction of* Atox1*, which encodes a chaperone that delivers copper to ATP7 transporters located in the trans-Golgi network [104], and* dCutC,* encoding a protein involved in the uptake, storage, delivery, and efflux of copper [105], suppressed both FRDA phenotypes. We also found that the* Metal-Responsive Transcription Factor-1* Gene* (MTF-1)* is a modifier of the motor impairment phenotype, acting as a suppressor when overexpressed and as an enhancer when downregulated. Overexpression of* MTF-1* in* Drosophila* also reduces the toxicity associated with oxidative stress [106], human A*β*42 peptide expression [107], and a parkin null mutation [108]. Under stress conditions, such as metal overload and oxidative stress, MTF-1 is translocated to the nucleus and binds to metal response elements (MREs) in the regulatory regions of its target genes, such as metal-sequestering metallothioneins (Mtns). Mtns are small cysteine-rich proteins that maintain low levels of intracellular free metal due to their ability to bind metals with high affinity. Contrary to what was expected, Mtn knockdown suppressed FRDA phenotypes [82], which could be explained by the role of Mtns as prooxidants under oxidative stress conditions [109–111]. Therefore, the beneficial effect of* MTF-1* overexpression may not be mediated by Mtns but rather by reduced iron accumulation, because the iron level is normalized in* fh*RNAi flies with* MTF-1* overexpression [82]. These results demonstrate that metal dysregulation in FRDA affects other metals in addition to iron. Importantly, zinc and copper redistribution have been reported in the dentate nucleus of the cerebellum in FRDA patients [112].

The genetic screen conducted in Calap-Quintana et al. [81] revealed four modifiers of the motor performance phenotype in FRDA flies. These genes encode tuberous sclerosis complex protein 1* (Tsc1), *ribosomal protein S6 kinase* (S6k)*, eukaryotic translation initiation factor 4E* (eIF-4F)*, and leucine-rich repeat kinase* (Lrrk)*. These proteins are involved in the TORC1 signaling pathway, which regulates many major cellular functions such as protein synthesis, lipid biogenesis, and autophagy. We found that genetic reduction in TORC1 signaling activity is beneficial, while its genetic activation produces a detrimental effect in frataxin knockdown flies by inducing semilethality.  Table 3 shows these genetic mediators of frataxin deficiency as well as other modifiers individually identified in other studies.

## 7. Potential Therapeutic Compounds for FRDA Treatment

Currently, there is no effective treatment for FRDA, although different therapeutic strategies are being developed or tested in clinical trials (http://www.curefa.org/pipeline). These strategies include lowering oxidative damage, reducing iron-mediated toxicity, increasing antioxidant defense, and increasing frataxin expression and gene therapy [83, 113, 114].* Drosophila* models are also gaining increasing significance in biomedical and pharmaceutical research as a valuable tool for testing potential treatments (Figures 4(b) and 4(c)).

Table 4 lists the compounds that have been found to improve some FRDA phenotypes in* Drosophila*. Our group has validated the utility of frataxin-depleted flies for drug screening [49]. We separately tested the effect of two compounds, the iron chelator deferiprone (DFP) and the antioxidant idebenone (IDE), that were already in use in clinical trials for this disease. DFP is a small-molecule, blood-brain-barrier-permeable drug that preferentially binds iron and prevents its reaction with ROS. IDE is a synthetic analog of coenzyme Q10 and can undergo reversible redox reactions, improving electron flux along the ETC. Each drug was administered in the fly food at two starting points: early treatment (from larva to adult stage) and adult treatment (in adult phase). Both drugs improved the lifespan and motor ability of flies expressing the* fh*-RNAi allele in a ubiquitous pattern or in the PNS* (neur)*, especially when given at the early treatment timepoint. DFP improved the FRDA phenotypes by sequestering mitochondrial iron and preventing toxicity induced by iron accumulation. IDE rescued aconitase activity in flies subjected to external oxidative stress [49].

Another compound with electron carrier properties, methylene blue (MB), has been described as a potent therapeutic drug for heart dysfunction in FRDA [42]. Cardiac defects were decreased in a dose-dependent manner in flies with heart-specific frataxin depletion treated with different concentrations of MB. The authors demonstrated that this drug was also able to reduce heart dilatation associated with deficiencies in several components of complexes I and III in mutant flies. These results indicate that respiratory chain impairment is involved in the cardiac defects associated with frataxin deficiency and that compounds showing electron transfer properties could prevent heart dysfunction in FRDA patients.

A yeast/*Drosophila* screen to identify new compounds for FRDA treatment was carried out by Seguin et al. [88]. The authors showed the utility of using a strategy based on two complementary models, a unicellular and a multicellular organism. Accordingly, a frataxin-deleted yeast strain was used in a primary screen, and positive hits were tested in flies ubiquitously expressing the UDIR2 allele (secondary screen). Approximately 6380 compounds were evaluated from two chemical libraries (the French National Chemical Library and the Prestwick Collection) to test the ability of the drugs to improve the fitness of yeast mutants using raffinose as the main carbon source. Yeast cells with frataxin deficiency grew slowly when raffinose was provided as the carbon source [115]. A total of 12 compounds, representative of the different chemical families, were selected from the yeast-based screen and their effect was analyzed on the FRDA fly model. Six of them improved the pupariation impairment of flies, with LPS 01-04-LG10 and Deferoxamine B (DFOB) being the most promising compounds. DFOB, an iron chelator, was suggested to increase the pools of bioavailable iron and to reduce iron accumulation in mitochondria. LPS 01-04-L-G10, a cinnamic derivative, partially rescued heart dilatation in flies with heart-specific frataxin depletion [88].

The efficacy of iron chelators as potential treatments has already been assessed in FRDA patients, but unfortunately the results were not conclusive. Studies have reported improvement of the cardiac and/or neurological conditions [61, 116, 117], no significant effect [118], or even worsening of some conditions [119]. However, the* Drosophila* models of FRDA indicate that iron is an important factor in FRDA pathophysiology. Genetic or pharmacological interventions through pathways regulating iron homeostasis and the sphingolipid/Pdk1/Mef-2 pathway are new approaches that might be explored in preclinical studies. In addition,* Drosophila* has shown for the first time that alteration of genes involved in metal detoxification and metal homeostasis (copper and zinc in addition to iron) is also a potential therapeutic strategy.

Finally, the results obtained from the genetic screen in* Drosophila* [81] also suggest that rapamycin and its analogs (rapalogs) are promising molecules for FRDA treatment. Inhibition of TORC1 signaling by rapamycin increases climbing speed, survival, and ATP levels in flies [81]. This compound enhances antioxidant defenses in both control and FRDA flies by increasing the nuclear translocation of the transcription factor encoded by the gene* cap-n-collar*, the* Drosophila* ortholog of* Nrf2*. As a result, it induces the expression of a battery of antioxidant genes. In addition, rapamycin protects against external oxidative stress by inducing autophagy. Rapamycin is a well-described drug approved for human uses. There is a large amount of data regarding the safety, tolerability, and side effects of this drug and rapalogs, which could facilitate their potential use in FRDA.

## 8. Conclusions

*D. melanogaster* is one of the most studied organisms in biological research. The conservation of many cellular and organismal processes between humans and flies and the constant increase in the number of genetic tools for* Drosophila* have made this organism one of the best choices for studying human genetic diseases. Following the identification of Friedreich's ataxia gene by positional cloning, model organisms have played a decisive role in the investigation of the function of frataxin and consequently the underlying pathophysiological mechanisms of FRDA. Here, we have presented the main contributions of* Drosophila* in this area of research. Frataxin-depleted flies recapitulate important biochemical, cellular, and physiological hallmarks of FRDA. In addition, the model flies exhibit new phenotypes that reveal, for the first time, other key players in FRDA pathogenesis. These models have allowed the identification of genetic and pharmacological factors capable of modifying some FRDA phenotypes, revealing new and promising ways to find effective treatments. Nevertheless, there are still many other questions that can be addressed by taking advantage of* Drosophila* models. Additional models of FRDA in flies are expected to help us understand the transcriptional silencing of* FXN *mediated by the GAA repeat expansion. These new models will advance our knowledge of the molecular bases of this disease and facilitate the development of new drugs for FRDA.

## Figures and Tables

**Table 1:** * Drosophila* models of frataxin deficiency. The *fh* construct or allele and the GAL4 driver used to obtain the different phenotypes of frataxin reduction are specified.

RNAi/mutant allele	GAL4 driver line	Phenotypes
*UDIR1*–*3* [33] frataxin reduction to undetectable levels (25°C)^*∗*^	*da* ^G32^ Ubiquitous	(i) Prolonged larval stages, reduced larvae viability, and inability to pupate [33, 88] (ii) When raised at 18°C, survivor adults exhibit high initial mortality, with some escapers that survive up to 40 days [33, 83] (iii) Reduction of activity of aconitase and respiratory complexes II, III, and IV in larvae and adults [33] (iv) Increase in free fatty acid content in larvae [83]
	*C96* Adult peripheral nervous system	(i) Viable adults with a shortened lifespan and increased sensitivity to H_2_O_2_ [33, 41]* *
	*D42* Motor neurons and interneurons in L3. Adult motor neurons	(i) Normal development and longevity [33] (ii) Loss of mitochondrial membrane potential and reduced mitochondrial transport in the distal axons. Distal axonal degeneration and cell body loss in the ventral ganglion in late L3 [48] (iii) Normal ROS levels [48]
	*Repo* Pan-glial	(i) Viable adults accompanied by some preadult lethality [83] (ii) Reduction of lifespan, increased sensitivity to hyperoxia (99.5% O_2_), and impaired climbing capability [66, 83] (iii) Lipid droplet accumulation in glial cells and brain vacuolization [66, 83]
	*HandGS* Heart-specific RU486-inducible Geneswitch driver	(i) Induction starting at L3. Viable adults that display heart dilatation and impaired systolic function [42, 88]
	*GMR* Developing eye	(i) Mild rough eye phenotype [82]

*UAS-fhIR* [34]: Up to 70% frataxin reduction (25°C)^*∗*^	*actin* and *da*^G32^ Ubiquitous	(i) Lethal at the mature pupa stage at 29°C [34] (ii) Viable adults that exhibit shortened lifespan, sensitivity to oxidative stress, and reduced climbing ability [34, 49, 66, 81, 82] (iii) Exposure to hyperoxia causes a substantial reduction in aconitase activity and oxygen consumption [34, 81, 83] (iv) Increased levels of lipid peroxides [81–83] (v) Increased mitochondrial iron content [49] (vi) Sensitive to increased iron content in diet [66] (vii) Complete ablation of iron-dependent ferritin accumulation, reduction of *IRP-1A* expression, and enhanced expression levels of *mfrn (mitoferrin)* [66] (viii) Increased levels of Fe, Zn, Cu, Mn, and Al [82]
	*neur* Sensory organs and their precursors	(i) Viable adults at 29°C [34] (ii) Reduced lifespan and climbing capability at 25 and 29°C [34, 49]
	*Nervous system:* *D42*, motor neurons *Ddc*, aminergic neurons and* c698a*, brain	(i) Viable adults at 29°C [34] (ii) Lifespan and climbing capability unaffected at 29°C [34]
	*Repo* Pan-glial.	(i) Viable adults [83] (ii) Reduction of lifespan, increased sensitivity to hyperoxia (99.5% O_2_), and impaired climbing capability [66, 83]
	*Other tissues:* *Dot*, heart and *24B*, mesoderm	(i) Lethal at the mature pupa stage at 29°C [34]

*fh* ^*1*^ [45]: Ethyl-methanesulfonate-induced missense mutation (S136R). Severe loss of *fh* function Mosaic *fh* mutant clones of adult photoreceptor neurons by the eyeless-FLP/FRT system		(i) Hemizygous *fh*^*1*^ mutants are lethal from L3 to pupa stage [45] (ii) Removal of maternal *fh* mRNA or protein in the egg causes embryonic lethality [45] (iii) Age-dependent degeneration of photoreceptors [45] (iv) Abnormal mitochondrial cristae morphology, reduced ETC CI activity, and impaired ATP production [45] (v) No increase in ROS [45] (vi) Accumulation of Fe^2+^ and/or Fe^3+^ and iron-dependent stimulation of sphingolipid synthesis and activation of the Pdk1/Mef2 pathway [45]

^*∗*^The most used temperature in the different experiments.

**Table 2:** Frataxin overexpression in *Drosophila. *The *fh* construct and the GAL4 driver used to obtain the different phenotypes are indicated.

Overexpression line	GAL4 driver line	Phenotypes
*UAS-dfh* ^*1*^ and *UAS-dfh*^*2*^ [89]: fourfold increase in *fh* mRNA expression (25°C)^*∗*^	*Actin* Ubiquitous	(i) Viable adults [89] (ii) Increased lifespan [89] (iii) Significant increase in tolerance to iron-induced stress (FeCl_3_), paraquat, and H_2_O_2_ (measuring survival) [89] (iv) Significant increase in total antioxidant activity (bathocuproine dye) [89]

*UAS-fh *[34]: 9-fold increase in *fh* mRNA expression and a strong increase in protein levels (29°C)^*∗*^	*Actin* and *da*^G32^ Ubiquitous	(i) Lethal at early pupae or 3rd instar larvae at 29°C [34] (ii) Defects in developing muscles, axonal tracks, and axonal pathfinding (1D4 staining) and an increase in the number of sensory ventral neurons. No abnormalities detected in the CNS [34] (iii) At 25°C, viable adults that are sensitive to oxidative stress and iron [34, 96]. Young individuals have higher catalase and aconitase activities and ATP production than controls but are hypersensitive to hyperoxia [96]
	*Appl *and *elav* Pan-neural	(i) Viable at 29°C and 25°C (ii) Reduced lifespan and climbing capability [95, 96]. Locomotor defects are rescued by mitochondrial catalase expression and *mfrn* silencing [96]. (iii) Reduced ferritin and mitoferrin levels [96] (iv) Brain vacuolization [96]
	*Other neuronal drivers* *neur* Sensory organs and their precursors *D42* Motor neurons *Ddc* Aminergic neurons *TH* Dopaminergic neurons *c698a* Brain	(i) Viable adults at 29°C and 25°C [34] (ii) Reduced climbing capability and lifespan at both temperatures *(neur/D42)* [34, 95]. (iii) Lifespan is recovered by mitochondrial catalase* (neur)* [95] (iv) *Ddc*, *TH*, and *c698a*: lifespan and climbing capability unaffected at 29°C or 25°C [34, 96] (v) Strong promotion of mitochondrial fusion and ROS-mediated cell death of dopaminergic neurons *(TH)* [96]
	*Repo* Pan-glial	(i) Reduced lifespan and climbing capability [95] (ii) Expression of mitochondrial catalase increases lifespan and climbing capability [95]
	*Other tissues:* *Dot*, heart and *24B*, mesoderm	(i) Lethal from the early pupa stage to adult eclosion from the puparium at 29°C and 25°C [34, 95] (ii) Lack of some pericardial cells along the tubular structure of the developing heart (ECII staining) in embryos at 29°C [34]

*UAS-FXN* ^#^ [95]: Expression of human frataxin. Stronger phenotypes than UAS-*fh* (25°C)^*∗*^	*Actin* and *da*^G32^ Ubiquitous	(i) Lethal in pupae [95] (ii) Reduced aconitase activity in larvae [95] (iii) Reduced NDUFS3 protein levels in larvae [95]
	*Appl* Pan-neural	(i) Viable adults, lethal at 29°C [95]
	*neur* Sensory organs and their precursors	(i) Reduced lifespan and climbing capability and increased sensitivity to oxidative insult [95] (ii) Expression of mitochondrial catalase increases lifespan [95]
	*Repo* Pan-glial	(i) Morphological disruption of glial cells and formation of lipid droplets [95] (ii) Expression of mitochondrial catalase increases lifespan and improves climbing capability [95]
	*24B* Mesoderm	(i) Lethal during pupariation [95]

^*∗*^The most used temperature in the experiments. ^#^UAS-*FXN* triggers the same defects as UAS-*fh*. To avoid repetition, only new phenotypes have been included; CNS: Central Nervous System.

**Table 3:** Genetic modifiers of FRDA phenotypes in *Drosophila*.

Modifier	Pathway	Effect
*Fer1HCH/Fer2LCH* (Co-expression)	Iron storage	Suppressor of reduced life span [66], ERG, and photoreceptor neurodegeneration [45]

*Fer3HCH* (OE)	Iron storage and oxidative stress protection	Suppressor of reduced life span [66] ERG, and photoreceptor neurodegeneration [45]

*Irp-1A* (RNAi) *Irp-1B* (RNAi) *Irp-1B* (LOF)	Iron sensor	Suppressor of mild rough eye and impaired motor performance [82]

*mfrn* (RNAi)	Mitochondrial iron importer	Suppressor of reduced aconitase activity and IRP-1A and ferritin levels, impaired motor performance, and increased brain vacuolization [66]
*mfrn* (OE)		Enhancer of locomotor defects and brain vacuolization [66]

*Mvl *(RNAi)	Iron absorption	Suppressor of mild rough eye and impaired motor performance [82]

*Tsf1 *(LOF) *Tsf3 *(RNAi)	Serum iron binding transport proteins	Suppressor of mild rough eye and impaired motor performance [82]

*dZip42C.1* (RNAi) *dZip42C*.2 (RNAi) *dZip88E* (RNAi)	Zinc importer	Suppressor of mild rough eye and impaired motor performance [82]

*dZnT35C* (RNAi)	Zinc transporter to vesicles	Suppressor of mild rough eye and impaired motor performance [82]

*dZnT41F* (RNAi)	Zinc homeostasis	Suppressor of mild rough eye and impaired motor performance [82]

*dZnT63C* (RNAi)	Zinc exporter	Suppressor of mild rough eye and impaired motor performance [82]

*foi* (LOF)	Zinc importer	Suppressor of impaired motor performance [82]

*Atox1* (RNAi)	Copper chaperone donor	Suppressor of mild rough eye and impaired motor performance [82]

*dCutC *(RNAi)	Copper uptake and storage	Suppressor of mild rough eye and impaired motor performance [82]

*MTF*-1 (OE)	Metal responsive Transcription Factor	Suppressor of impaired motor performance [82]
*MTF-1* (LOF)		Enhancer of impaired motor performance [82]

*MtnA* (RNAi)	Heavy metal detoxification	Suppressor of mild rough eye and impaired motor performance [82]

*MtnB* (RNAi) *MtnC* (RNAi)	Heavy metal detoxification	Suppressor of mild rough eye [82]

*Tsc1* (RNAi)	TORC1 pathway	Enhancer of reduced survival [81]

*S6K* (DN)	TORC1 pathway	Suppressor of impaired motor performance [81]
*S6K* (CA)		Enhancer of reduced survival [81]

*eIF-4E* (LOF)	TORC1 pathway	Suppressor of impaired motor performance [81]

*Lrrk* (RNAi)	TORC1 pathway	Suppressor of impaired motor performance [81]

*Cat* (OE) *mCat* (OE) *mTPx* (OE)	Antioxidant (hydrogen peroxide scavengers)	Suppressor of reduced lifespan when overexpressed in the PNS [41]

*dGLaz* (OE)	Antioxidant defense	Suppressor of reduced life span, impaired motor performance, aconitase inactivation, and lipid peroxidation [83]

*Pdk1* (RNAi)	Embryonic development (insulin receptor transduction pathway and apoptotic pathway)	Suppressor of photoreceptor neurodegeneration [45]

*Mef2* (RNAi)	Muscle differentiation	Suppressor of photoreceptor neurodegeneration [45]

*lace* (RNAi)	Sphingosine biosynthesis pathway	Suppressor of photoreceptor neurodegeneration [45]

CA: constitutively active mutation; DN: dominant negative mutation; ERG: electroretinograms; LOF: loss-of-function mutation; OE: overexpression; RNAi: RNA interference.

**Table 4:** Compounds that showed beneficial effects in *Drosophila* models of FRDA.

Compound	Mechanism of action	Improved phenotype
Idebenone	Antioxidant	Motor performance and lifespan in adults [42, 49]
Methylene blue	Electron carrier	Adult heart function [42]
Toluidine blue	Electron carrier	Adult heart function [42]
Deferiprone	Iron chelator	Motor performance and lifespan in adults [49]
Deferoxamine	Iron chelator	Pupa development [88]
LPS 01-03-L-F03	Possible iron chelator	Pupa development [88]
LPS 02-25-L-E10	Possible iron chelator	Pupa development [88]
LPS 02-13-L-E04	Possible iron chelator	Pupa development [88]
LPS 01-04-L-G10	n.d.	Pupa development [88] Adult heart function [88]
LPS 02-14-L-B11	n.d.	Pupa development [88]
Rapamycin	TORC1 inhibitor	Motor performance and oxidative stress in adults [81]
Myriocin	Serine palmitoyltransferase inhibitor	Photoreceptor function [45]

n.d.: not described.

## References

[1] Harding A. (1993). Clinical features and classification of inherited ataxias. *Advances in Neurology*.

[2] Bidichandani S. I., Delatycki M. B. Friedreich Ataxia.

[3] Campuzano V., Montermini L., Moltò M. D. (1996). Friedreich's ataxia: autosomal recessive disease caused by an intronic GAA triplet repeat expansion. *Science*.

[4] Koutnikova H., Campuzano V., Foury F., Dollé P., Cazzalini O., Koenig M. (1997). Studies of human, mouse and yeast homologues indicate a mitochondrial function for frataxin. *Nature Genetics*.

[5] Koutnikova H., Campuzano V., Koenig M. (1998). Maturation of wild-type and mutated frataxin by the mitochondrial processing peptidase. *Human Molecular Genetics*.

[6] Condò I., Ventura N., Malisan F., Rufini A., Tomassini B., Testi R. (2007). In vivo maturation of human frataxin. *Human Molecular Genetics*.

[7] Campuzano V., Montermini L., Lutz Y. (1997). Frataxin is reduced in Friedreich ataxia patients and is associated with mitochondrial membranes. *Human Molecular Genetics*.

[8] Galea C. A., Huq A., Lockhart P. J. (2016). Compound heterozygous FXN mutations and clinical outcome in friedreich ataxia. *Annals of Neurology*.

[9] Le S. Q., Gascuel O. (2010). Accounting for solvent accessibility and secondary structure in protein phylogenetics is clearly beneficial. *Systematic Biology*.

[10] Kumar S., Stecher G., Tamura K. (2016). MEGA7: Molecular Evolutionary Genetics Analysis version 7.0 for bigger datasets. *Molecular Biology and Evolution*.

[11] Kelley L. A., Mezulis S., Yates C. M., Wass M. N., Sternberg M. J. E. (2015). The Phyre2 web portal for protein modeling, prediction and analysis. *Nature Protocols*.

[12] Pettersen E. F., Goddard T. D., Huang C. C. (2004). UCSF Chimera—a visualization system for exploratory research and analysis. *Journal of Computational Chemistry*.

[13] Brand A. H., Perrimon N. (1993). Targeted gene expression as a means of altering cell fates and generating dominant phenotypes. *Development*.

[14] González-Cabo P., Vicente Llorens J., Palau F., Dolores Moltó M. (2009). Friedreich ataxia: An update on animal models, frataxin function and therapies. *Advances in Experimental Medicine and Biology*.

[15] Pandolfo M., Pastore A. (2009). The pathogenesis of Friedreich ataxia and the structure and function of frataxin. *Journal of Neurology*.

[16] Stemmler T. L., Lesuisse E., Pain D., Dancis A. (2010). Frataxin and mitochondrial FeS cluster biogenesis. *The Journal of Biological Chemistry*.

[17] Marmolino D. (2011). Friedreich's ataxia: Past, present and future. *Brain Research Reviews*.

[18] Busi M. V., Gomez-Casati D. F. (2012). Exploring frataxin function. *IUBMB Life*.

[19] Gomes C. M., Santos R. (2013). Neurodegeneration in Friedreich's ataxia: From defective frataxin to oxidative stress. *Oxidative Medicine and Cellular Longevity*.

[20] Vaubel R. A., Isaya G. (2013). Iron-sulfur cluster synthesis, iron homeostasis and oxidative stress in Friedreich ataxia. *Molecular and Cellular Neuroscience*.

[21] Pastore A., Puccio H. (2013). Frataxin: A protein in search for a function. *Journal of Neurochemistry*.

[22] Anzovino A., Lane D. J. R., Huang M. L.-H., Richardson D. R. (2014). Fixing frataxin: 'Ironing out' the metabolic defect in Friedreich's ataxia. *British Journal of Pharmacology*.

[23] Chiang S., Kovacevic Z., Sahni S. (2016). Frataxin and the molecular mechanism of mitochondrial iron-loading in Friedreich's ataxia. *Clinical Science*.

[24] Martelli A., Puccio H. (2014). Dysregulation of cellular iron metabolism in Friedreich ataxia: from primary iron-sulfur cluster deficit to mitochondrial iron accumulation. *Frontiers in Pharmacology*.

[25] Maio N., Rouault T. A. (2015). Iron-sulfur cluster biogenesis in mammalian cells: new insights into the molecular mechanisms of cluster delivery. *Biochimica et Biophysica Acta (BBA)—Molecular Cell Research*.

[26] Parent A., Elduque X., Cornu D. (2015). Mammalian frataxin directly enhances sulfur transfer of NFS1 persulfide to both ISCU and free thiols. *Nature Communications*.

[27] Santos R., Lefevre S., Sliwa D., Seguin A., Camadro J.-M., Lesuisse E. (2010). Friedreich ataxia: molecular mechanisms, redox considerations, and therapeutic opportunities. *Antioxidants & Redox Signaling*.

[28] Bayot A., Rustin P. (2013). Friedreich's ataxia, frataxin, PIP5K1B: Echo of a distant fracas. *Oxidative Medicine and Cellular Longevity*.

[29] González-Cabo P., Palau F. (2013). Mitochondrial pathophysiology in Friedreich's ataxia. *Journal of Neurochemistry*.

[30] Reiter L. T., Potocki L., Chien S., Gribskov M., Bier E. (2001). A systematic analysis of human disease-associated gene sequences in *Drosophila melanogaster*. *Genome Research*.

[31] Fortini M. E., Skupski M. P., Boguski M. S., Hariharan I. K. (2000). A survey of human disease gene counterparts in the *Drosophila* genome. *The Journal of Cell Biology*.

[32] Cañizares J., Blanca J. M., Navarro J. A., Monrós E., Palau F., Moltó M. D. (2000). dfh is a Drosophila homolog of the Friedreich's ataxia disease gene. *Gene*.

[33] Anderson P. R., Kirby K., Hilliker A. J., Phillips J. P. (2005). RNAi-mediated suppression of the mitochondrial iron chaperone, frataxin, in *Drosophila*. *Human Molecular Genetics*.

[34] Llorens J. V., Navarro J. A., Martínez-Sebastían M. J. (2007). Causative role of oxidative stress in a *Drosophila* model of Friedreich ataxia. *The FASEB Journal*.

[35] Kondapalli K. C., Kok N. M., Dancis A., Stemmler T. L. (2008). Drosophila frataxin: An iron chaperone during cellular Fe-S cluster bioassembly. *Biochemistry*.

[36] Yoon T., Cowan J. A. (2004). Frataxin-mediated iron delivery to ferrochelatase in the final step of heme biosynthesis. *The Journal of Biological Chemistry*.

[37] Dzul S. P., Rocha A. G., Rawat S. (2017). In vitro characterization of a novel Isu homologue from Drosophila melanogaster for de novo FeS-cluster formation. *Metallomics*.

[38] Marelja Z., Leimkühler S., Missirlis F. (2018). Iron Sulfur and Molybdenum Cofactor Enzymes Regulate the Drosophila Life Cycle by Controlling Cell Metabolism. *Frontiers in Physiology*.

[39] Perdomini M., Hick A., Puccio H., Pook M. A. (2013). Animal and cellular models of Friedreich ataxia. *Journal of Neurochemistry*.

[40] Kennerdell J. R., Carthew R. W. (2000). Heritable gene silencing in Drosophila using double-stranded RNA. *Nature Biotechnology*.

[41] Anderson P. R., Kirby K., Orr W. C., Hilliker A. J., Phillips J. P. (2008). Hydrogen peroxide scavenging rescues frataxin deficiency in a *Drosophila* model of Friedreich's ataxia. *Proceedings of the National Acadamy of Sciences of the United States of America*.

[42] Tricoire H., Palandri A., Bourdais A., Camadro J.-M., Monnier V. (2014). Methylene blue rescues heart defects in a *Drosophila* model of Friedreich's ataxia. *Human Molecular Genetics*.

[43] Osterwalder T., Yoon K. S., White B. H., Keshishian H. (2001). A conditional tissue-specific transgene expression system using inducible GAL4. *Proceedings of the National Acadamy of Sciences of the United States of America*.

[44] Roman G., Endo K., Zong L., Davis R. L. (2001). P{switch}, a system for spatial and temporal control of gene expression in drosophila melanogaster. *Proceedings of the National Acadamy of Sciences of the United States of America*.

[45] Chen K., Lin G., Haelterman N. A. (2016). Loss of frataxin induces iron toxicity, sphingolipid synthesis, and Pdk1/Mef2 activation, leading to neurodegeneration. *eLife*.

[46] Bridwell-Rabb J., Fox N. G., Tsai C.-L., Winn A. M., Barondeau D. P. (2014). Human frataxin activates Fe-S cluster biosynthesis by facilitating sulfur transfer chemistry. *Biochemistry*.

[47] Cossée M., Puccio H., Gansmuller A. (2000). Inactivation of the Friedreich ataxia mouse gene leads to early embryonic lethality without iron accumulation. *Human Molecular Genetics*.

[48] Shidara Y., Hollenbeck P. J. (2010). Defects in mitochondrial axonal transport and membrane potential without increased reactive oxygen species production in a Drosophila mdel of Friedreich aaxia. *The Journal of Neuroscience*.

[49] Soriano S., Llorens J. V., Blanco-Sobero L. (2013). Deferiprone and idebenone rescue frataxin depletion phenotypes in a Drosophila model of Friedreich's ataxia. *Gene*.

[50] Sanchez-Casis G., Cote M., Barbeau A. (1976). Pathology of the Heart in Friedreich's Ataxia: Review of the Literature and Report of One Case. *Canadian Journal of Neurological Sciences / Journal Canadien des Sciences Neurologiques*.

[51] Lamarche J. B., Côté M., Lemieux B. (1980). The Cardiomyopathy of Friedreich's Ataxia Morphological Observations in 3 Cases. *Canadian Journal of Neurological Sciences / Journal Canadien des Sciences Neurologiques*.

[52] Foury F., Cazzalini O. (1997). Deletion of the yeast homologue of the human gene associated with Friedreich's ataxia elicits iron accumulation in mitochondria. *FEBS Letters*.

[53] Michael S., Petrocine S. V., Qian J. (2006). Iron and iron-responsive proteins in the cardiomyopathy of Friedreich's ataxia. *The Cerebellum*.

[54] Ramirez R. L., Qian J., Santambrogio P., Levi S., Koeppen A. H. (2012). Relation of cytosolic iron excess to cardiomyopathy of friedreich's ataxia. *American Journal of Cardiology*.

[55] Koeppen A. H., Ramirez R. L., Becker A. B. (2015). The pathogenesis of cardiomyopathy in Friedreich ataxia. *PLoS ONE*.

[56] Waldvogel D., Van Gelderen P., Hallett M. (1999). Increased iron in the dentate nucleus of patients with Friedreich's ataxia. *Annals of Neurology*.

[57] Bradley J. L., Blake J. C., Chamberlain S., Thomas P. K., Cooper J. M., Schapira A. H. V. (2000). Clinical, biochemical and molecular genetic correlations in Friedreich's ataxia. *Human Molecular Genetics*.

[58] Koeppen A. H., Michael S. C., Knutson M. D. (2007). The dentate nucleus in Friedreich's ataxia: The role of iron-responsive proteins. *Acta Neuropathologica*.

[59] Koeppen A. H., Morral J. A., Davis A. N. (2009). The dorsal root ganglion in Friedreich's ataxia. *Acta Neuropathologica*.

[60] Harding I. H., Raniga P., Delatycki M. B. (2016). Tissue atrophy and elevated iron concentration in the extrapyramidal motor system in Friedreich ataxia: The IMAGE-FRDA study. *Journal of Neurology, Neurosurgery & Psychiatry*.

[61] Boddaert N., Sang K. H. L. Q., Rötig A. (2007). Selective iron chelation in Friedreich ataxia: Biologic and clinical implications. *Blood*.

[62] Chen K., Ho T. S.-Y., Lin G., Tan K. L., Rasband M. N., Bellen H. J. (2016). Loss of frataxin activates the iron/sphingolipid/PDK1/Mef2 pathway in mammals. *eLife*.

[63] Puccio H., Simon D., Cossée M. (2001). Mouse models for Friedreich ataxia exhibit cardiomyopathy, sensory nerve defect and Fe-S enzyme deficiency followed by intramitochondrial iron deposits. *Nature Genetics*.

[64] Simon D., Seznec H., Gansmuller A. (2004). Friedreich Ataxia Mouse Models with Progressive Cerebellar and Sensory Ataxia Reveal Autophagic Neurodegeneration in Dorsal Root Ganglia. *The Journal of Neuroscience*.

[65] Al-Mahdawi S., Pinto R. M., Varshney D. (2006). GAA repeat expansion mutation mouse models of Friedreich ataxia exhibit oxidative stress leading to progressive neuronal and cardiac pathology. *Genomics*.

[66] Navarro J. A., Botella J. A., Metzendorf C., Lind M. I., Schneuwly S. (2015). Mitoferrin modulates iron toxicity in a *Drosophila* model of Friedreich's ataxia. *Free Radical Biology & Medicine*.

[67] Rotig A., de Lonlay P., Chretien D. (1997). Aconitase and mitochondrial iron-sulphur protein deficiency in friedreich ataxia. *Nature Genetics*.

[68] Lynch Gwen Lech D. R., Farmer J. M., Balcer L. J., Bank W., Chance B., Wilson R. B. (2002). Near infrared muscle spectroscopy in patients with Friedreich's ataxia. *Muscle & Nerve*.

[69] Palandri A., L'hôte D., Cohen-Tannoudji J., Tricoire H., Monnier V. (2015). Frataxin inactivation leads to steroid deficiency in flies and human ovarian cells. *Human Molecular Genetics*.

[70] Moreno-Cermeño A., Obis È., Bellí G., Cabiscol E., Ros J., Tamarit J. (2010). Frataxin depletion in yeast triggers up-regulation of iron transport systems before affecting iron-sulfur enzyme activities. *The Journal of Biological Chemistry*.

[71] Armstrong J. S., Khdour O., Hecht S. M. (2010). Does oxidative stress contribute to the pathology of Friedreich's ataxia? A radical question. *The FASEB Journal*.

[72] Bulteau A.-L., Dancis A., Gareil M., Montagne J.-J., Camadro J.-M., Lesuisse E. (2007). Oxidative stress and protease dysfunction in the yeast model of Friedreich ataxia. *Free Radical Biology & Medicine*.

[73] Vázquez-Manrique R. P., González-Cabo P., Ros S., Aziz H., Baylis H. A., Palau F. (2006). Reduction of Caenorhabditis elegans frataxin increases sensitivity to oxidative stress, reduces lifespan, and causes lethality in a mitochondrial complex II mutant. *The FASEB Journal*.

[74] Wong A., Yang J., Cavadini P. (1999). The Friedreich's ataxia mutation confers cellular sensitivity to oxidant stress which is rescued by chelators of iron and calcium and inhibitors of apoptosis. *Human Molecular Genetics*.

[75] Santos R., Buisson N., Knight S. A. B., Dancis A., Camadro J.-M., Lesuisse E. (2004). Candida albicans lacking the frataxin homologue: A relevant yeast model for studying the role of frataxin. *Molecular Microbiology*.

[76] Busi M. V., Maliandi M. V., Valdez H. (2006). Deficiency of Arabidopsis thaliana frataxin alters activity of mitochondrial Fe-S proteins and induces oxidative stress. *The Plant Journal*.

[77] Condo I., Ventura N., Malisan F., Tomassini B., Testi R. (2006). A pool of extramitochondrial frataxin that promotes cell survival. *The Journal of Biological Chemistry*.

[78] Napoli E., Taroni F., Cortopassi G. A. (2006). Frataxin, iron-sulfur clusters, heme, ROS, and aging. *Antioxidants & Redox Signaling*.

[79] Irazusta V., Moreno-Cermeño A., Cabiscol E., Ros J., Tamarit J. (2008). Major targets of iron-induced protein oxidative damage in frataxin-deficient yeasts are magnesium-binding proteins. *Free Radical Biology & Medicine*.

[80] Rustin P., Von Kleist-Retzow J.-C., Chantrel-Groussard K., Sidi D., Munnich A., Rötig A. (1999). Effect of idebenone on cardiomyopathy in Friedreich's ataxia: A preliminary study. *The Lancet*.

[81] Calap-Quintana P., Soriano S., Llorens J. V. (2015). TORC1 inhibition by rapamycin promotes antioxidant defences in a drosophila model of friedreich's ataxia. *PLoS ONE*.

[82] Soriano S., Calap-Quintana P., Llorens J. V. (2016). Metal homeostasis regulators suppress FRDA phenotypes in a drosophila model of the disease. *PLoS ONE*.

[83] Navarro J. A., Ohmann E., Sanchez D. (2010). Altered lipid metabolism in a *Drosophila* model of Friedreich's ataxia. *Human Molecular Genetics*.

[84] Abeti R., Uzun E., Renganathan I., Honda T., Pook M. A., Giunti P. (2015). Targeting lipid peroxidation and mitochondrial imbalance in Friedreich's ataxia. *Pharmacological Research*.

[85] Martelli A., Friedman L. S., Reutenauer L. (2012). Clinical data and characterization of the liver conditional mouse model exclude neoplasia as a non-neurological manifestation associated with Friedreich's ataxia. *Disease Models & Mechanisms*.

[86] Worth A. J., Basu S. S., Deutsch E. C. (2015). Stable isotopes and LC-MS for monitoring metabolic disturbances in Friedreich's ataxia platelets. *Bioanalysis*.

[87] Tamarit J., Obis È., Ros J. (2016). Oxidative stress and altered lipid metabolism in Friedreich ataxia. *Free Radical Biology & Medicine*.

[88] Seguin A., Monnier V., Palandri A. (2015). A Yeast/Drosophila Screen to Identify New Compounds Overcoming Frataxin Deficiency. *Oxidative Medicine and Cellular Longevity*.

[89] Runko A. P., Griswold A. J., Min K.-T. (2008). Overexpression of frataxin in the mitochondria increases resistance to oxidative stress and extends lifespan in Drosophila. *FEBS Letters*.

[90] Schulz T. J., Westermann D., Isken F. (2010). Activation of mitochondrial energy metabolism protects against cardiac failure. *AGING*.

[91] Miranda C. J., Santos M. M., Ohshima K., Tessaro M., Sequeiros J., Pandolfo M. (2004). Frataxin overexpressing mice. *FEBS Letters*.

[92] Ristow M., Pfister M. F., Yee A. J. (2000). Frataxin activates mitochondrial energy conversion and oxidative phosphorylation. *Proceedings of the National Acadamy of Sciences of the United States of America*.

[93] Shoichet S. A., Bäumer A. T., Stamenkovic D. (2002). Frataxin promotes antioxidant defense in a thiol-dependent manner resulting in diminished malignant transformation in vitro. *Human Molecular Genetics*.

[94] Schulz T. J., Thierbach R., Voigt A. (2006). Induction of oxidative metabolism by mitochondrial frataxin inhibits cancer growth: Otto Warburg revisited. *The Journal of Biological Chemistry*.

[95] Navarro J. A., Llorens J. V., Soriano S. (2011). Overexpression of human and fly frataxins in drosophila provokes deleterious effects at biochemical, physiological and developmental levels. *PLoS ONE*.

[96] Edenharter O., Clement J., Schneuwly S., Navarro J. A. (2017). Overexpression of Drosophila frataxin triggers cell death in an iron-dependent manner. *Journal of Neurogenetics*.

[97] Martelli A., Schmucker S., Reutenauer L. (2015). Iron Regulatory Protein 1 Sustains Mitochondrial Iron Loading and Function in Frataxin Deficiency. *Cell Metabolism*.

[98] Mühlenhoff U., Gerber J., Richhardt N., Lill R. (2003). Components involved in assembly and dislocation of iron-sulfur clusters on the scaffold protein Isu1p. *EMBO Journal*.

[99] Froschauer E. M., Schweyen R. J., Wiesenberger G. (2009). The yeast mitochondrial carrier proteins Mrs3p/Mrs4p mediate iron transport across the inner mitochondrial membrane. *Biochimica et Biophysica Acta (BBA) - Biomembranes*.

[100] Brazzolotto X., Pierrel F., Pelosi L. (2014). Three conserved histidine residues contribute to mitochondrial iron transport through mitoferrins. *Biochemical Journal*.

[101] Liuzzi J. P., Aydemir F., Nam H., Knutson M. D., Cousins R. J. (2006). Zip14 (Slc39a14) mediates non-transferrin-bound iron uptake into cells. *Proceedings of the National Acadamy of Sciences of the United States of America*.

[102] Wang C.-Y., Jenkitkasemwong S., Duarte S. (2012). ZIP8 is an iron and zinc transporter whose cell-surface expression is up-regulated by cellular iron loading. *The Journal of Biological Chemistry*.

[103] Xiao G., Wan Z., Fan Q., Tang X., Zhou B. (2014). The metal transporter ZIP13 supplies iron into the secretory pathway in Drosophila melanogaster. *eLife*.

[104] Walker J. M., Tsivkovskii R., Lutsenko S. (2002). Metallochaperone Atox1 transfers copper to the NH2-terminal domain of the Wilson's disease protein and regulates its catalytic activity. *The Journal of Biological Chemistry*.

[105] Li Y., Du J., Zhang P., Ding J. (2010). Crystal structure of human copper homeostasis protein CutC reveals a potential copper-binding site. *Journal of Structural Biology*.

[106] Bahadorani S., Mukai S., Egli D., Hilliker A. J. (2010). Overexpression of metal-responsive transcription factor (MTF-1) in Drosophila melanogaster ameliorates life-span reductions associated with oxidative stress and metal toxicity. *Neurobiology of Aging*.

[107] Hua H., Münter L., Harmeier A., Georgiev O., Multhaup G., Schaffner W. (2011). Toxicity of Alzheimer's disease-associated A*β* peptide is ameliorated in a Drosophila model by tight control of zinc and copper availability. *biological chemistry*.

[108] Saini N., Georgiev O., Schaffner W. (2011). The parkin mutant phenotype in the fly is largely rescued by metal-responsive transcription factor (MTF-1). *Molecular and Cellular Biology*.

[109] Fabisiak J. P., Pearce L. L., Borisenko G. G. (1999). Bifunctional Anti-/Prooxidant Potential of Metallothionein: Redox Signaling of Copper Binding and Release. *Antioxidants & Redox Signaling*.

[110] Suntres Z. E., Lui E. M. K. (2006). Prooxidative effect of copper-metallothionein in the acute cytotoxicity of hydrogen peroxide in Ehrlich ascites tumour cells. *Toxicology*.

[111] Suzuki K. T., Rui M., Ueda J.-I., Ozawa T. (1996). Production of hydroxyl radicals by copper-containing metallothionein: Roles as prooxidant. *Toxicology and Applied Pharmacology*.

[112] Koeppen A. H., Ramirez R. L., Yu D. (2012). Friedreich's ataxia causes redistribution of iron, copper, and zinc in the dentate nucleus. *The Cerebellum*.

[113] Wilson R. B. (2012). Therapeutic developments in Friedreich ataxia. *Journal of Child Neurology*.

[114] Richardson T. E., Kelly H. N., Yu A. E., Simpkins J. W. (2013). Therapeutic strategies in Friedreich's ataxia. *Brain Research*.

[115] Lesuisse E., Santos R., Matzanke B. F., Knight S. A. B., Camadro J.-M., Dancis A. (2003). Iron use for haeme synthesis is under control of the yeast frataxin homologue (Yfh1). *Human Molecular Genetics*.

[116] Velasco-Sánchez D., Aracil A., Montero R. (2011). Combined therapy with idebenone and deferiprone in patients with Friedreich's ataxia. *The Cerebellum*.

[117] Elincx-Benizri S., Glik A., Merkel D. (2016). Clinical Experience with Deferiprone Treatment for Friedreich Ataxia. *Journal of Child Neurology*.

[118] Arpa J., Sanz-Gallego I., Rodríguez-de-Rivera F. J. (2014). Triple therapy with deferiprone, idebenone and riboflavin in Friedreich's ataxia - open-label trial. *Acta Neurologica Scandinavica*.

[119] Pandolfo M., Arpa J., Delatycki M. B. (2014). Deferiprone in Friedreich ataxia: A 6-month randomized controlled trial. *Annals of Neurology*.

